# NMDA and Dopamine Converge on the NMDA-Receptor to Induce ERK Activation and Synaptic Depression in Mature Hippocampus

**DOI:** 10.1371/journal.pone.0000138

**Published:** 2006-12-27

**Authors:** Hanoch Kaphzan, Kenneth J. O'Riordan, Kile P. Mangan, Jonathan M. Levenson, Kobi Rosenblum

**Affiliations:** 1 Center for Brain and Behavior, Department of Neurobiology and Ethology, Haifa University, Haifa, Israel; 2 Department of Pharmacology and the Waisman Center, University of Wisconsin School of Medicine and Public Health, Madison, Wisconsin, United States of America; University of Washington, United States of America

## Abstract

The formation of enduring internal representation of sensory information demands, in many cases, convergence in time and space of two different stimuli. The first conveys the sensory input, mediated via fast neurotransmission. The second conveys the meaning of the input, hypothesized to be mediated via slow neurotransmission. We tested the biochemical conditions and feasibility for fast (NMDA) and slow (dopamine) neurotransmission to converge on the Mitogen Activated Protein Kinase signaling pathways, crucial in several forms of synaptic plasticity, and recorded its effects upon synaptic transmission. We detected differing kinetics of ERK2 activation and synaptic strength changes in the CA1 for low and high doses of neurotransmitters in hippocampal slices. Moreover, when weak fast and slow inputs are given together, they converge on ERK2, but not on p38 or JNK, and induce strong short-term synaptic depression. Surprisingly, pharmacological analysis revealed that a probable site of such convergence is the NMDA receptor itself, suggesting it serves as a detector and integrator of fast and slow neurotransmission in the mature mammalian brain, as revealed by ERK2 activation and synaptic function.

## Introduction

The decision-making regarding the meaning of a given sensory input is hypothesized to be dependent upon “heterosynaptic modulation” [1]. In general, two types of synapses are involved: ionotropic and metabotropic (also called “fast” and “slow”). The “dialogue” between these different types of synapses, along with their reciprocal interactions, is referred as heterosynaptic modulation [1]. One example of heterosynaptic facilitation in the nervous system is the interaction between ionotropic glutamate receptors and metabotropic dopamine receptors in the mature hippocampus [2], [3].

In the hippocampus, heterosynaptic modulation is involved in Long-Term Potentiation (LTP) and consolidation of long-term memory. Several studies have demonstrated that heterosynaptic modulation facilitates the induction of NMDA-dependent early and late-phases of LTP via tetanizing stimuli in area CA1 of the hippocampus [4], [5], [6], [7]. In addition, blocking dopaminergic neurotransmission prevents or attenuates late-phase LTP [1], [4], [6]. Moreover, strong dopaminergic input by itself can induce LTP in the absence of tetanizing stimulation [4].

Modulation of signal transduction pathways is an attractive mechanism for heterosynaptic intonation of synaptic plasticity. Many signal transduction pathways have been implicated in the induction of synaptic plasticity in the hippocampus, and many of these pathways are interconnected, providing several opportunities for modulation. Thus, certain signal transduction molecules could serve as “coincidence detectors” of sensory information and its value, and would facilitate memory consolidation [8]. For example, a diminutive and insignificant sensory input might converge on the signal transduction induced as a result of the saliency of another input, resulting in enhanced activation of downstream signaling pathways and eventually result in induction of synaptic plasticity and/or consolidation of long-term memory.

Numerous signaling molecules could potentially serve as a read out of coincidence detectors, but prominent in neurons are those that comprise the Mitogen Activated Protein Kinase (MAPK) signaling cascades, which include several distinct kinases that are critical for normal neuronal function [9]. They include the Extracellular signal Regulated-Kinases (ERKs), c-Jun-N-terminal kinases (JNKs) and p38 MAPK [10]. Among these, ERK1/2 occupies a unique place in the hippocampus as it has been implicated in behavioral memory, long-lasting synaptic plasticity, and biochemical information processing at the molecular level [8], [10]. Specifically, ERK is involved in both early- and late-phase hippocampal LTP [11], [12], and a number of different learning paradigms, including fear conditioning and spatial learning (Sweatt, 2004).

Surprisingly, the convergent activation of MAPKs by dopamine and NMDA has not been studied systematically *in vitro* or *in vivo*, especially given that the temporal pattern of MAPK activation is commonly used as a crucial parameter for the readout cellular responses [13], [14], and up today, though suggested, no study has solidly supported the hypothesis of MAPKs as potential fast and slow coincident detectors. That is not to rule out other molecules, acting upstream to MAPKs, that might serve as coincident detectors, such as adenylyl-cyclases [15], [16]. The current study is unique in its examination of the convergence of NMDA and dopamine-mediated signaling in a direct pharmacological stimulation paradigm (not a dopaminomimetic agent), employing biochemical and extra-cellular electrophysiological essays, while emphasizing the time dependency of the combined stimuli, the possible molecular mechanism of such convergence, the site accountable for it, and its expression through MAPK's cascades.

In the present study, we determined the dose and temporal activation patterns of ERK2 activation by each neurotransmitter alone and simultaneously. We then tested the hypothesis that members of the MAPK family serve as coincidence detectors of NMDA and dopaminergic signaling in the mature brain. In exploring this hypothesis, we discovered that regulation of ERK phosphorylation behaves as a possible cellular site of NMDA-dopamine signaling convergence. Moreover, we examined the effect of these two neurotransmitters, in the various concentrations upon the synaptic transmission in the CA1. In agreement with the molecular analysis, electrophysiological analysis revealed that NMDA and dopamine in low doses induced different effects then high doses, and that the co-application converges on the NR to induce strong short-term synaptic depression. Our results suggest a molecular framework that explains how the similarity in consolidation processes between strong sensory input alone and convergence of multiple weak sensory inputs.

## Results

### Hippocampal slice preparation and MAPK measurements

For *in vitro* testing of the hypothesis that dopamine and NMDA converge on the MAPK signaling cascade in the mature brain, we used pharmacological manipulation of mature hippocampal slices in an ACSF-perfused interface chamber (see Materials and Methods, also[17]). In agreement with previous findings, we detected increased ERK1/2 activation immediately following slice preparation [18]. This increase was accompanied by variability in ERK1/2 activation, which returned to baseline levels 4–5 h following slice preparation (data not shown). A physiological interpretation of the importance of this prolonged incubation period has been previously discussed [19]. Therefore, all of our experiments were done after slices were incubated for 5 h.

Following the calibration experiments, we first tested activation of ERK2 by application of a high dose of NMDA (100 µM) with 10 µM glycine [20], [21]. In agreement with previous observations [20], we detected significant and transient ERK2 activation by 100 µM NMDA, which peaked at 5 min (2.14±0.09, n = 8) relative to the control (1±0.06, n = 8). However, the activation was not affected by pretreatment with and co-application of 1 µM TTX (2.11±0.16, n = 8), demonstrating that ERK2 activation by NMDA and glycine is due specifically to activation of NMDA-Rs and not to NMDA-induced synaptic activity or increase in excitability.

### Dose dependency of ERK2 activation by NMDA and dopamine

To test the effect of dopaminergic heterosynaptic modulation upon NMDA neurotransmission we determined the concentrations of dopamine and NMDA that elicited the minimum detectable activation of ERK2, and also the exact timing of activation.

**Figure 1:**
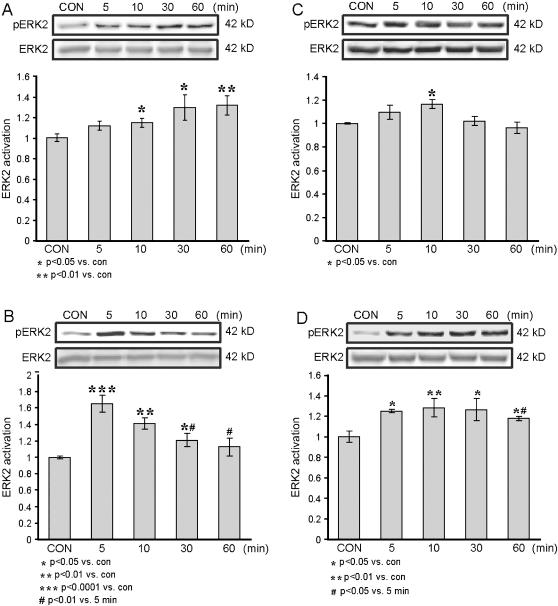
Time dependency of ERK2 activation by NMDA or dopamine. Western blot analysis of the time dependency of ERK2 activation, as measured by the ratio of pERK2/ERK2, in response to NMDA or dopamine application to hippocampal slices. NMDA was co-applied with 10 µM glycine, and dopamine was co-applied with 1 mM ascorbic acid within various time frames. Hippocampal slices were harvested in the course of the various time frames, snap frozen on dry ice, homogenized in SDS sample buffer, and subjected to Western blot analysis of the different specimens. Antibodies against the phosphorylated ERK2 were directed to phosphorylation sites Thr183/Tyr185 (here and below). Each specimen comprised two hippocampal slices combined (i.e., 2 slices is n = 1) (this applies to all the following figure captions). A. NMDA was co-applied with glycine, each at a concentration of 10 µM (n = 6 for each time frame). B. NMDA was co-applied with glycine at concentrations of 100 and 10 µM respectively (n = 6 for each time frame). C. dopamine at a concentration 10 µM was co-applied with 1 mM ascorbic acid (n = 6 for each time frame). D. Dopamine at a concentration 100 µM was co-applied with 1 mM ascorbic acid (n = 6 for each time frame). Here and after representative immunoblots are depicted above the graphs.

Application of 10 µM NMDA resulted in a gradual increase in phosphorylation of ERK2; activation was not significant at 5 min of NMDA application (1.11±0.04, *p* = 0.06, n = 6), but was significant at 10, 30 and 60 min (1.15±0.04, *p*<0.05, n = 6; 1.28±0.11, *p*<0.05, n = 6; and 1.31±0.09, *p*<0.01, n = 6, respectively). The control level was 1±0.02, n = 6. Also, in the first 60 min of NMDA application there was no dip in the ERK2 activation level (Fig. 1A).

Application of 100 µM NMDA resulted in a different temporal pattern of ERK2 activation, which was maximal after 5 min of NMDA application (1.66±0.11, *p*<0.0001, n = 6), and decayed rapidly. Activation of ERK2 was still significant 10 and 30 min after NMDA application compared to control (1.42±0.07, *p*<0.01, n = 6; 1.21±0.08, *p*<0.05, n = 6, respectively). By 60 minutes of application, phosphorylation of ERK2 was not significantly different from the control level (1.12±0.11, *p* = 0.3, n = 6; control, 1±0.01, n = 6) (Fig. 1B). Moreover, at 30 and 60 min of NMDA application the levels of P-ERK2 were significantly lower than those measured after 5 min (*p*<0.01). Thus, weak activation of NMDA-receptors (NMDA-R) induced a sustained increase in pERK2 while strong activation of NMDA-Rs induced a robust, but transient activation of ERK2. This pattern of NMDA-induced regulation of ERK is consistent with previously published findings [20], [21].

Application of 10 µM dopamine resulted in a weak and transient ERK2 activation, which peaked within 10 min (1.17±0.04, *p*<0.05, n = 6) and then decayed to levels no different from that of the control (1±0.006, n = 6) (Fig. 1C). Application of 100 µM dopamine resulted in a faster and more sustained activation of ERK2, which approached its peak within 5 min (1.25±0.02, *p*<0.05, n = 6), and plateau at 10 and 30 min (1.28±0.09, *p*<0.01, n = 6; 1.26±0.11, *p*<0.05, n = 6). After 60 min of 100 µM dopamine application, ERK2 activation remained significantly different from control (1.18±0.02, *p*<0.05, n = 6; 1±0.05, n = 6, respectively), but had decayed to a level significantly less than the 5 min peak (*p*<0.05) (Fig. 1D). Thus, weak dopaminergic signaling induced a transient increase in pERK2 while strong dopaminergic signaling induced a sustained activation of ERK2.

### ERK2 activation kinetics under co-application of dopamine and NMDA

Strong activation of ERK has been implicated in induction of synaptic plasticity and consolidation of long-term memory. We hypothesized that heterosynaptic modulation at the level of ERK would result from two, weak signals converging synergistically to induce robust activation of ERK. Therefore, we decided to look for convergence of NMDA and dopamine signaling specifically at the low doses of these compounds, as convergence upon ERK would likely be easier to detect.

**Figure 2:**
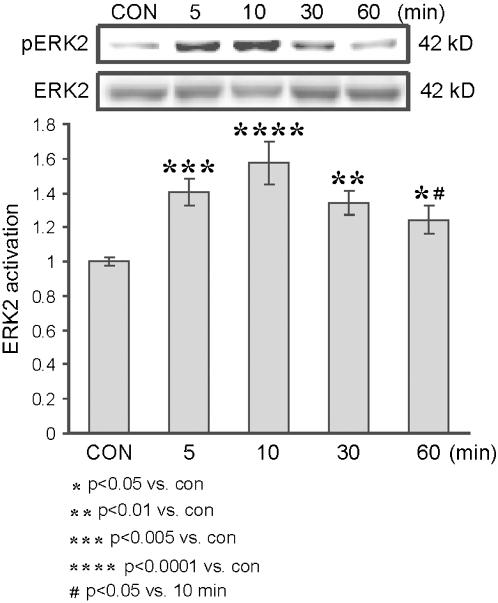
Time dependency of ERK2 activation following co-application of low doses of dopamine and NMDA. Western blot analysis of time dependency of ERK2 activation, as measured by the ratio of pERK2/ERK2, in response to co-application of dopamine and NMDA to hippocampal slices. Dopamine at 10 µM was co-applied with 1 mM ascorbic acid, 10 µM NMDA and 10 µM glycine for the different time frames (n = 6 for each time frame).

We first analyzed the time dependency of ERK2 activation associated with the convergence of the low doses (10 µM) of dopamine and NMDA. The pattern of ERK2 activation found in response to this co-stimulation was distinctive both in time dependency and magnitude: activation was faster and stronger than that obtained with a low concentration of either NMDA or dopamine. Moreover, the magnitude and kinetics of NMDA-dopamine co-application were similar to the application of high dose of NMDA (figure1B). Co-application of NMDA and dopamine elicited significant activation of ERK2 (1.41±0.08, *p*<0.005, n = 6) within 5 min, activation peaked within 10 min (1.58±0.12, *p*<0.0001, n = 6), and began to decay within 30 min (1.34±0.07, *p*<0.01, n = 6). However, even at 60 min the phosphorylation of ERK remained significantly higher than that of the control (1.24±0.08, *p*<0.05, n = 6; 1.0±0.02, n = 6, respectively) (Fig. 2).

### Dopamine and NMDA stimulation converge on ERK2 but not p38 nor JNK

**Figure 3:**
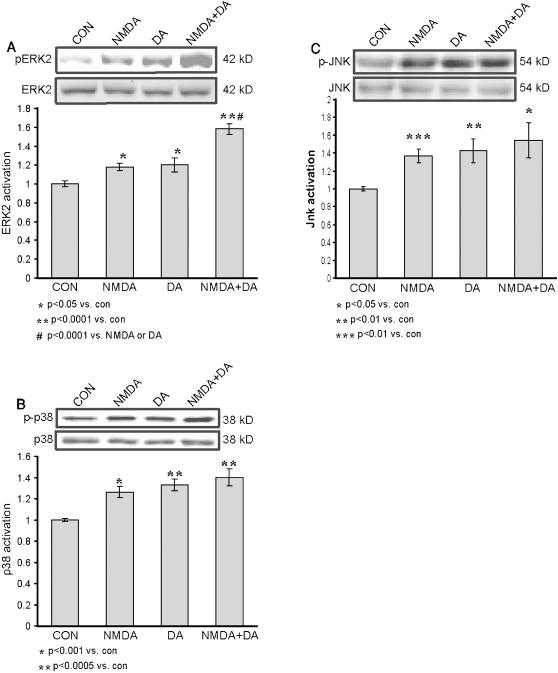
ERK2 but neither p38 nor JNK serves as a coincidence detector of dopamine and NMDA application. Western blot analysis of MAPKs activation, as measured by the ratio of phospho-MAPK/total MAPK, induced by 10 min application of either 10 µM NMDA with 10 µM glycine (n = 6), or of 10 µM dopamine with 1 mM ascorbic acid (n = 6), or co-application of 10 µM dopamine, 1 mM ascorbic acid, 10 µM NMDA and 10 µM glycine (n = 6), or control (n = 6). A. ERK2 activation; B. p38 activation. Antibodies against the phosphorylated p38 were directed to phosphorylation sites Thr180/Tyr182; C. JNK activation. Antibodies against the phosphorylated JNK were directed to phosphorylation sites Thr183/Tyr185.

In conclusion, it appeared that the convergence of NMDA and dopamine, when given in low doses, resulted in a faster and stronger activation of ERK2 than that obtained with either separately. Because of slight differences shown to be among different batches of slices, in order to determine whether the convergence was stronger within the same batch of hippocampal slices, we compared the ERK2 activation levels within 10 min of application, in slices from the same batch only. In light of the kinetics shown in the previous experiments, we chose 10 min of application as the time frame within which to analyze the signal convergence. Slices were harvested after 10 min of transmitter application, and compared the activation levels in the controls, and in slices exposed to 10 µM NMDA and to 10 µM dopamine, separately and together. Co-application induced a significantly stronger activation of ERK2 than that induced by 10 µM dopamine or 10 µM NMDA alone. ERK2 activation by 10 µM NMDA was weak but significantly increased compare with the control (1.18±0.04, *p*<0.05, n = 6 and 1.0±0.02, n = 6, respectively). ERK2 activation by 10 µM dopamine was also slightly greater than that in the control, but significant (1.20±0.07, *p*<0.05, n = 6 and 1.0±0.02, n = 6, respectively) (Fig. 3A). ERK2 activation by the co-application of NMDA+dopamine, each at 10 µM was strong and significantly greater than that in the control (1.58±0.06, *p*<0.0001, n = 6 and 1±0.02, n = 6, respectively) (Fig. 3A). Moreover, it was also significantly greater than that caused by either 10 µM NMDA alone or 10 µM dopamine alone (*p*<0.0001 in both comparisons) (Fig. 3A). The MAPK signaling cascades include other members than ERK that are known to play roles in synaptic plasticity and long-term memory [22], [23], [24], [25], [26]. The question that was addressed in the present study was whether this pattern of signal transduction convergence is unique to ERK2, or whether it is common to other members of the MAPK signaling cascades, i.e., JNK and p38. Therefore, the same specimens that had been used in looking for convergence of the two agents in ERK2 activation were also analyzed for p38 and JNK activation.

Significant activation of p38 within 10 min was observed in response to separate application of either 10 µM NMDA or 10 µM dopamine (1.26±0.05, *p*<0.001, n = 6; 1.33±0.05, *p*<0.0005, n = 6, respectively), compared with that in the control (1.0±0.01, n = 6) (Fig. 3B). The p38 activation within 10 min of co-application was also significant in comparison with that in the control (1.40±0.08, *p*<0.0005, n = 6; and 1.0±0.01, n = 6, respectively) (Fig. 3B), but no significant convergence was observed in comparison with either agent alone (*p* = 0.18 vs. NMDA, *p* = 0.48 vs. dopamine) (Fig. 3B).

Analysis of the same specimens for JNK activation yielded similar results to those for p38 activation. Both 10 µM NMDA and 10 µM dopamine, acting separately, induced significant activation compared with that in the control within 10 min of application: (1.37±0.07, *p*<0.001, n = 6; 1.42±0.13, *p*<0.01, n = 6; and 1.0±0.02 n = 6, respectively) (Fig. 3C). Co-application of NMDA and dopamine induced a significant activation of JNK compared to control (1.54±0.19, *p*<0.05, n = 6), but no significant further augmentation was detected as a result of such co-application (*p* = 0.44 vs. NMDA, *p* = 0.63 vs. dopamine). Hence, no significant convergence of the effects of NMDA and dopamine on the activation of JNK was observed.

### Dopamine activation of ERK2 is NMDA receptor dependent, but NMDA activation of ERK2 is dopamine receptor independent

Thus far, our data indicate that ERK2 activation serves as a point of convergence readout for NMDA and dopaminergic neurotransmission. However, in order to better identify the upstream convergence site, we tested the hypothesis that the NMDA receptor itself serves as a coincident-detector of NMDA and dopamine neurotransmission. Several previous studies indicate that the effects of dopamine or dopamine agonists *in vitro* and *in vivo* require NMDA-mediated synaptic transmission [27], [28], [29], [30]. The NMDA-receptor serves as a classic example of convergence in the nervous system whereby NMDA-receptor—mediated synaptic transmission only occurs after removal of Mg^2+^ from its ion channel. Therefore, we tested the possibility that the NMDA-R itself can also act as a point of convergence with dopaminergic neurotransmission.

**Figure 4:**
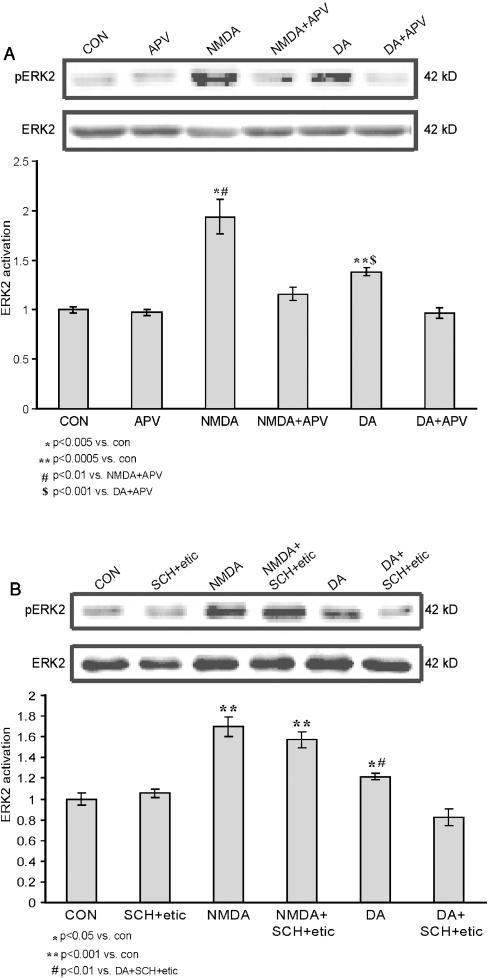
ERK2 activation by dopamine is NMDA receptor dependent, while ERK2 activation by NMDA is dopamine receptor independent. A. Western blot analysis of ERK2 activation, as measured by the ratio of pERK2/ERK2, induced by 10 min application of either 20 µM NMDA with 10 µM glycine with and without APV 40 µM (n = 4, each), or of 20 µM dopamine with 1 mM ascorbic acid with and without APV 40 µM (n = 4, each). Also shown are control (n = 4) and APV 40 µM applied alone for 30 minutes (n = 4). Each application of either dopamine or NMDA, when co-applied with APV, was pre-perfused with APV 40 µM alone for 30 minutes, prior to their co-application with APV. B. Western blot analysis of ERK2 activation, as measured by the ratio of pERK2/ERK2, induced by 10 min application of either 20 µM NMDA with 10 µM glycine with and without co-application of SCH23390 40 µM and eticlopride 60 µM (n = 4, each), or of 20 µM dopamine with 1 mM ascorbic acid with and without co-application of SCH23390 40 µM and eticlopride 60 µM (n = 4, each). Also shown are control (n = 4), and co-application of SCH23390 40 µM and eticlopride 60 µM for 30 minutes (n = 4). Each application of either dopamine or NMDA, when co-applied with SCH23390 and eticlopride, was pre-perfused with SCH23390 40 µM and eticlopride 60 µM for 30 minutes, prior to their co-application with these antagonists.

In order to get a significant and definite response with the various stimulations (since 10 µM is a threshold concentration for stimulation) we employed concentrations of 20 µM NMDA and dopamine each. Previous experiments observed that these concentrations are sufficient to yield maximal ERK2 activation. Both NMDA 20 µM and Dopamine 20 µM, applied separately, induced significant activation of ERK2 compared to control (1.93±0.17, *p*<0.005, n = 4; 1.38±0.04, *p*<0.0005, n = 4; 1±0.03, n = 6, respectively). Blocking the NMDA receptor with D-APV (D-2-amino-5-phosphonovaleric acid) 40 µM during NMDA 20 µM or dopamine 20 µM stimulations yields similar outcomes. While APV (40 µM) abolished the NMDA induced ERK2 activation as expected (1.16±0.07, n = 4, *p*<0.01 vs. NMDA, *p* = 0.09 vs. cont), it also completely abolished any dopamine induced ERK2 activation as well (0.97±0.05, n = 4, *p*<0.001 vs. dopamine, *p* = 0.61 vs. cont) (Fig. 4A). In order to examine a possible reciprocal effect of NMDA on dopamine receptors, we stimulated with NMDA and blocked dopamine receptors with a combination of SCH23390 (40 µM, D1/5 antagonist) co-applied with eticlopride (60 µM, D2 antagonist). Once again we employed concentrations of 20 µM NMDA and dopamine each, in order to get a significant response with the various stimulations. While dopamine 20 µM induced a significant ERK2 activation compared to control (1.22±0.03, *p*<0.05 n = 4; 1±0.06, n = 4, respectively), SCH23390 and eticlopride abolished the dopamine induced ERK2 activation as expected (0.83±0.08, n = 4; *p*<0.005 vs. dopamine; *p* = 0.13 vs. control). There was no effect of the SCH23390 and eticlopride on NMDA-induced activation of ERK. NMDA 20 µM induced a significant ERK2 activation compared to control (1.70±0.10, *p*<0.001 n = 4; 1±0.06, n = 4, respectively). SCH23390 co-applied with eticlopride during NMDA 20 µM stimulation induced significant ERK2 activation compared to control, which was not significantly different from activation of ERK2 in response to NMDA alone (1.57±0.08, n = 4; *p*<0.001 vs. control; *p* = 0.35 vs. NMDA), (Fig. 4B).

### Co-application of low doses of NMDA and dopamine has similar functional properties as strong NMDA activation on synaptic plasticity

**Figure 5:**
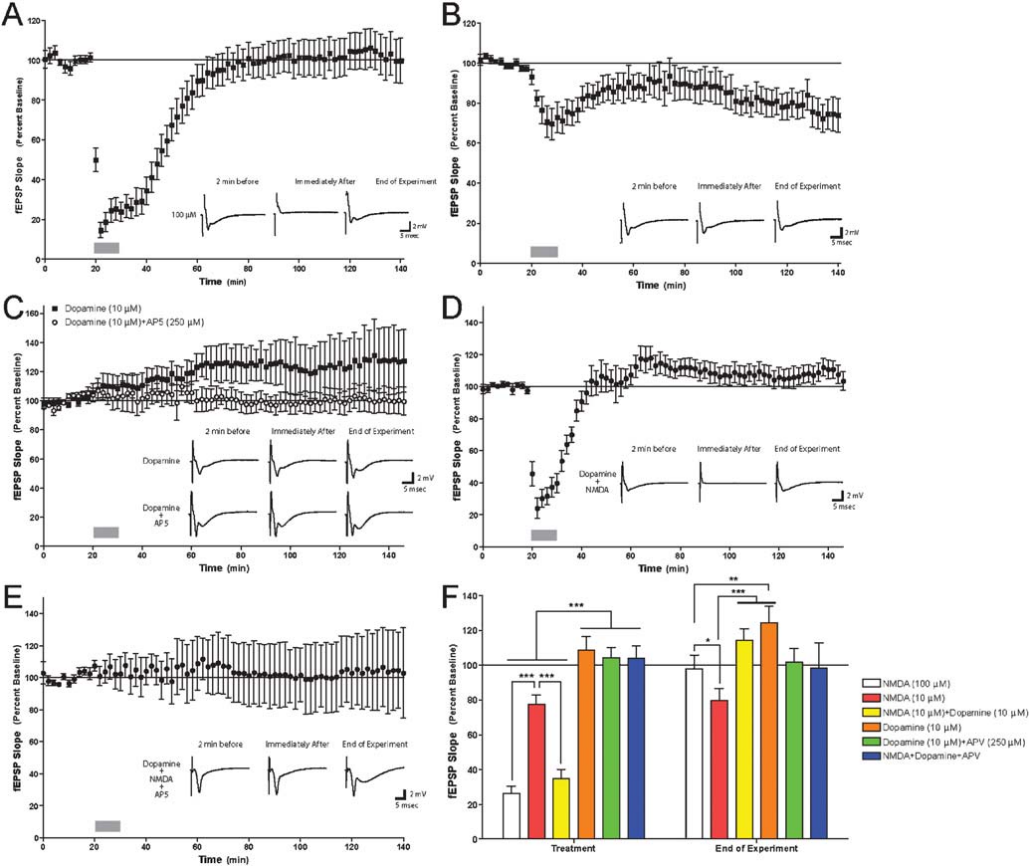
The effect of high and low doses of NMDA and dopamine on efficacy of Schaffer-collateral synapses. The effect of NMDA and dopamine on the efficacy of Schaffer-collateral synapses was determined. A. Strong NMDA stimulation (100 µM, 10 min) resulted in a robust and immediate depression of synaptic efficacy. After washout, synaptic efficacy returned to basal levels within 60 min of treatment (n = 21 slices). Grey bar indicates treatment. Inset illustrates representative traces 2 min before treatment, immediately after treatment and at the end of the experiment. B. Weak NMDA stimulation (10 µM, 10 min) resulted in a rapid depression of synaptic transmission that persisted for at least 120 min after treatment (n = 21 slices). C. Weak dopamine stimulation (10 µM, 10 min) resulted in a gradual potentiation of synaptic efficacy (n = 11 slices). The effect of weak dopamine stimulation was completely inhibited by the NMDA-R antagonist DL-APV (50 µM, n = 3 slices). D. Co-application of weak NMDA (10 µM) and dopamine (10 µM) resulted in a robust and immediate depression of synaptic efficacy. After washout, synaptic efficacy returned to basal levels within 60 min of treatment (n = 15 slices). Note the similarity with the effect of strong NMDA in panel A. E. The NMDA-R antagonist DL-APV (50 µM) completely inhibited the effect of co-application of weak NMDA (10 µM) and dopamine (10 µM) on synaptic efficacy (n = 4 slices). F. Summary statistics of the effect of treatments with neurotransmitters on Schaffer-collateral synaptic efficacy. ‘Baseline’ data were averaged over the first 20 min of recording where no treatment was administered. ‘Treatment’ data represent the average fEPSP during the 10 min where NMDA/Dopamine/APV were administered. ‘End of Experiment’ represents the average fEPSP from 130–140 min from the start of an experiment. No significant differences were observed between the experimental groups during the baseline. Significant differences across treatments were observed during treatment and at the end of experiment using pair-wise post-hoc Bonferroni comparisons. Asterisks indicate significant differences (* = p<0.05, ** = p<0.01, *** = p<0.001). In all panels, error bars indicate SEM.

The results above demonstrated that the molecular kinetics of ERK activation is similar between strong NMDA and co-application of weak NMDA and dopamine. We thus tested another hypothesis that strong activation of the NMDA-R will have similar functional effects on synaptic efficacy in the hippocampus as with co-application of low doses of NMDA and dopamine. Towards that end, we examined the effect of the various chemical stimulation protocols on synaptic efficacy at Schaffer-collateral synapses. Application of 100 µM NMDA for 10 minutes induced a sharp and transient fEPSP depression with a gradual return to baseline within 40 minutes (Fig. 5A, F; F_[20,70]_ = 13, p<0.0001). Application of NMDA 10 µM for 10 minutes induced long term depression (LTD) which lasted for 2 hours of recording (Fig. 5B, F; F_[20,70]_ = 1.5, p<0.005), while application of dopamine 10 µM for 10 minutes induced weak long-term potentiation (LTP) lasting for 2 hours of recording as well (Fig. 5C, F; F_[10,70]_ = 101, p<0.01). Co-application of NMDA and dopamine, 10 µM each, induced qualitatively similar acute and long-term responses as NMDA 100 µM (Fig.5D,F; F_[14,70]_ = 14, p<0.0001), indicating a similarity in physiological effect between the two stimulation protocols (Fig. 5F, fEPSP during depression [mean±SEM]: NMDA 100 µM – 26.2%±4.1%, NMDA 10 µM+Dopamine 10 µM – 34.8%±5.2%; t = 0.9, df = 34, p<0.4). Collectively, the effects either of low doses of NMDA or dopamine alone on synaptic efficacy were dramatically different (i.e. increase in fEPSP versus decrease in fEPSP, Fig. 5B, C, F). However application of low doses of NMDA and dopamine together had an effect on synaptic efficacy that yielded results similar to stimulation with strong NMDA input (figures 5A, D, F). These results are consistent with our observations that a combined, weak stimulation with NMDA and dopamine together have the same effect on ERK that strong NMDA stimulation elicits.

### Dopamine induced potentiation as well as NMDA and dopamine co-application induced depression is NMDA receptor dependent

Activation of ERK2 by dopamine was NMDA-R—dependent, suggesting that the effects of dopamine on the hippocampus were via the NMDA-R (Fig. 4A). To determine whether the effect of dopamine on synaptic efficacy was NMDA-R—dependent, we applied dopamine (10 µM) with the NMDA antagonist APV. APV inhibited the weak potentiation induced by dopamine alone (Fig. 5C, F; F_1,13_ = 41, p<0.0001), thus representing the crucial role for NMDA receptor as a mediator of the dopaminergic effect on synaptic efficacy. This also coincides with the biochemical hypothesis that dopamine affects ERK2 in the hippocampus via the NMDA-R. Either way, we can see that the dopaminergic effect is NMDA-R dependent. Moreover, APV also inhibited the strong depression induced by co-application of low NMDA and dopamine (Fig. 5D, E, F; F_[3,70]_ = 0.08, p = 1; fEPSP at time of depression [mean±SEM]: NMDA+Dopamine – 34.8%±5.2%, NMDA+Dopamine+APV – 104%±7.2%). This inhibition not only attenuated the synaptic response, but totally erased any synaptic change. That is to show that not only the low NMDA effect was abolished, but also the dopamine effect as well (Fig.5E). Once again this indicates the crucial and exclusive role of the NMDA-R both in integrating the NMDA and dopamine effect, as well as mediating the dopamine effect alone.

### The proposed mechanism for the synaptic depression in high dose NMDA and co-application of dopamine and NMDA low doses is via reduce expression of synaptosomal AMPA-R

**Figure 6:**
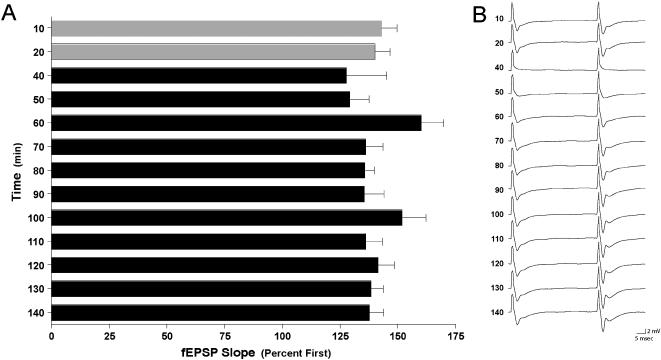
Strong NMDA treatments do not alter paired-pulse facilitation. Paired-pulse facilitation (PPF) at an inter-stimulus interval of 50 msec was measured in slices (n = 7) before and after treatment with NMDA (100 µM, 10 min). A. No significant differences were observed in PPF after treatment with NMDA (F_13,6_ = 1.3, p<0.3). Grey bars indicate PPF before treatment with NMDA. Black bars indicate PPF after treatment with NMDA. B. Representative traces illustrating PPF.

**Figure 7:**
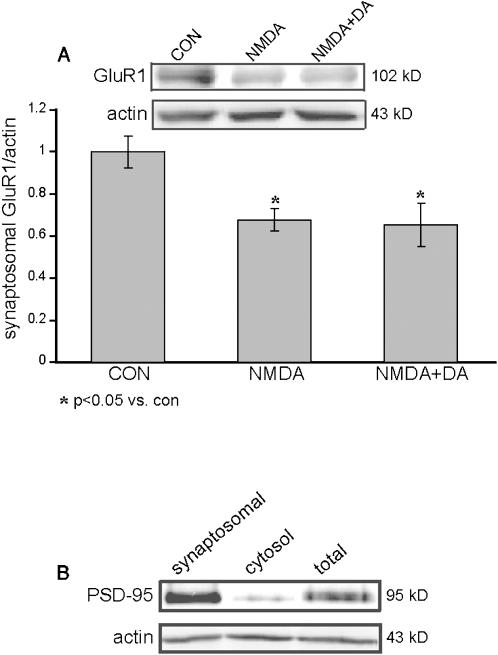
Application of 100 µM NMDA or co application of NMDA and dopamine, 10 µM each induces reduction in the synaptosomal GluR1 content. A. Western blot analysis of synaptosomal GluR1 and β-actin content, induced by 10 min application of either NMDA 100 µM with 10 µM glycine (n = 4), or co-application of 10 µM dopamine, 1 mM ascorbic acid, 10 µM NMDA and 10 µM glycine (n = 4), or control (n = 4). Synaptosomal fraction was performed as mentioned previously in the methods. Relative quantification of synaptosomal GluR1 content was measured in relation to β-actin within the same blot. B. Synaptosomal fraction validation. Western blot comparison of synaptosomal enriched fraction, non-synaptosomal fraction and total homogenate with PSD-95, as a synaptosomal marker, and β-actin within the same blot.

In order to examine further if the biological mechanisms subserving the short-term depression is shared between strong NMDA application and co-application of NMDA and dopamine (Fig. 5A, D, F), we measured PPF and the amount of synaptosomal GluR1 expression following the different treatments. There was no effect on PPF (Fig. 6A; F_13,6_ = 1.3, p<0.3) following stimulation with NMDA (100 µM), suggesting that the acute synaptic depression was due to regulation of post-synaptic processes, and not due to pre-synaptic ones. Indeed, when we examined the amount of synaptosomal GluR1 expression 10 minutes following treatment with strong NMDA and co-application of weak NMDA and dopamine groups, we observed a significant reduction in the amount of GluR1 in synaptosomes (1±0.07, n = 4; 0.67±0.05, *p*<0.05, n = 4; 0.65±0.1, *p*<0.05, n = 4; control, NMDA 100 µM and NMDA+dopamine 10 µM each respectively) (Fig. 7A). Synaptosomal enrichment was validated by western blot analysis of PSD-95 (Fig. 7B).

## Discussion

Slow neuromodulatory and fast neurotransmission are considered to converge on molecules within a given neuron to produce the heterosynaptic modulation phenomenon. The hypothesis that the two converge on MAPK pathways and synaptic function in mature hippocampal slices was tested in the current study. Classically, adenylyl cyclases (ACs), especially AC1 and AC8 are proposed to serve as molecular coincident detectors in this convergence phenomena [15]. However, the plot thickened when it was recently shown that even in AC1-AC8 double knockout hippocampal neurons, NMDA induces calcineurin dependent cAMP increase, suggesting other AC active at that locus [31]. MAPK pathways which are downstream to ACs are also hypothesized to serve such a role as well [8]. In our view the readout of convergence on the MAPKs level might be a mere expression of the convergence on the ACs. The various MAPKs, especially ERK2 and p38 are known to be activated by ACs, and in rare cases (though sometimes reciprocal) even JNK. Therefore the downstream readout of the convergence on the MAPKs level is important in deciphering the cellular response. To investigate this question we employed hippocampal slices pharmacological essay. This method enabled us to investigate this phenomenon in a reduced preparation derived from mature brain that conserves in part their connectivity, while exerting maximal control upon the pharmacological stimulation. MAPK plays a major role in diverse cellular and neuronal activities, and its activation is necessary for consolidation of long-term memory and LTP (for review see [32]). Regulation of MAPK activity occurs through various mechanisms, including phosphorylation, duration of activation and subcellular localization [33]. At the receptor level, ERK1/2 is activated by either NMDA or dopamine [20], [34], [35], [36], [37], [38], [39], [40], [41], [42]. Application of glutamate to hippocampal cell cultures induces an NMDA-R -dependent increase in ERK1/2 phosphorylation [34], [43]. ERK1/2 is also activated through NMDA-dependent electrophysiological stimulating potentiation protocols [35], and by NMDA application upon hippocampal slices [20], [36]. However, the time-variation of ERK1/2 activation has been investigated only at high doses (100 µM), where NMDA-induced activation of ERK was fast and transient: significant in 2.5 min, maximal at 5 min, and decaying within 10 min. Longer periods were not investigated [20], and neither was the time-variation of the activation of ERK1/2 by low doses of NMDA. As in the case of NMDA, application of dopamine upon neural cell cultures results in ERK1/2 activation [37], [39]. The same holds for cultures of other types of cells, such as alveolar epithelial cells [38]. ERK1/2 activation patterns were individually shown for D1 [39], as well as for D2 receptors [40], though for D1 receptors there is conflicting evidence regarding ERK1/2 activation [41], [42]. Dose dependency of dopamine stimulation has been investigated in a neural cell culture, and was found to be maximal at a concentration of 100 µM [39], and examination of the time course of ERK1/2 activation at this dopamine concentration revealed a prolonged activation that was maximal within 45 min and remained significant for 2 h. Neither the time course of low-dose dopamine stimulation, nor that of the effects of dopamine application upon brain slices has been investigated.

Other members of the MAPK family (e.g., JNK and p38) are activated by NMDA and dopamine. JNK is activated by dopamine D1 [41], [44], D2 [45] and by NMDA [46], [47], [48]. p38 is activated by dopamine D1 [39], [41], [42] and by NMDA in a complex manner [49]. In certain conditions D2 stimulation did not activate p38 [41]. Nevertheless, it was recently shown that dopamine itself, and either D1 agonist or D2 agonist, all induce various MAPKs activation [50]. In spite of this research, coincidence detection between NMDA and dopamine signaling has not been determined in any of the MAPK family members.

In order to test the possibility that MAPK might serve as a coincidence detector of NMDA and dopamine inputs, we first established time and dose curves for NMDA and for dopamine. In addition, we determined the minimal doses of NMDA and of dopamine that induced MAPK activation. We observed that there were differences, not only in the magnitude of the activation but also in its kinetics (Fig. 1). ERK2 activation by 100 µM NMDA exhibited maximal activation at 5 min, and a fast decay, so that at 30 min the decay is significant compared to the 5 min peak, and to near baseline levels within 60 min (Fig. 1).

The same pattern of activation, up to 10 min, was reported by [20], and the differences in the magnitude of activation caused by the various concentrations were also previously reported, in part [21]. In the present study, such a decay pattern was not observed with 10 µM NMDA but, rather a pattern of consistent activation that developed with time, reached significance within 30 min, and did not decay by 60 min. The difference among the activation patterns elicited by the various NMDA doses can be attributed to NMDA receptor desensitization, or, in the case of 100 µM NMDA, to strong activation of phosphatases, which would result in rapid dephosphorylation of ERK2, thereby lowering its level within the cell. Alternatively, the difference might be attributed to the activation of different signaling pathways with differing kinetics, especially regarding their affinities to the various NMDA receptor subtypes, e.g., the differences seen with NR1-NR2B and NR1-NR2A activation [51], [52], [53]. NR1-NR2B is considered to be a receptor with a higher affinity for agonists than NRI-NR2A and, though both concentrations – 10 µM and 100 µM – are above the maximal affinity values, the actual concentration within the synapse is not known, so that this difference in NMDA concentration might cause a significant difference in subunit activation [54], [55], [56]. The difference between the effects of dopamine at 10 and 100 µM was smaller than that observed with different doses of NMDA. The lower dopamine dose caused a weak transient activation that might be regarded as a weaker activation than that caused by the high dose, so that it was a transitory effect, but the difference might also be accounted for by different signaling pathways or activation of different receptor subtypes.

The essential role of dopaminergic heterosynaptic modulation in determining NMDA-dependent input, and its implications have already been addressed in the past by various methods. Some studies applied tetanization stimulation, which was NMDA-dependent, while blocking or stimulating dopaminergic receptors, either D1 or D2 separately, in analyzing the effect upon synaptic potentiation [2], [4], [57]. Another study employed dihydrexidine (D1/5 agonist) stimulation of cultured hippocampal neurons while NMDA receptors were blocked with APV, and found that the surface GluR1 immunoreactivity and colocalization with synaptophysin was reduced, and the mEPSC frequency was inhibited [27], thus exhibiting a cellular outcome for this ambiguous form of transactivation between these two types of receptors.

Though, the possibility of coincidence detection at the level of signal transduction has been raised previously, and was especially supported for adenylyl-cyclases [15], [16], the downstream molecular patterns involved in this putative converging activation is unknown, and has never been proven in a direct activation by NMDA and dopamine. In particular, all of the research regarding detection of coincident NMDA and dopamine stimulation of ERK1/2 activation employed indirect dopaminergic stimulation with cocaine, while NMDA receptors were blocked [29], [30], or stimulation with D1R agonist while blocking NMDA receptors [28]. We thus set up to investigate this interaction in a direct manner in hippocampal slice preparation.

After we had determined 10 min to be the time of peak activation by low doses of NMDA and dopamine, we investigated this activation mode further in additional experiments. ERK2 activation time dependent curve was measured as a response to the mutual NMDA+dopamine co-application. That experiment was performed in order to locate a suitable time frame for examining the possible convergence effect. In response to this co-application, ERK2 activation had a bursting pattern of fast and strong development within 5 min, maximal at 10 min, and eventually a tendency to decay at 60 min, a pattern which resembles the activation pattern in high dose NMDA application (Fig. 3). Accordingly, 10 min was found to be the preferred time frame for the further investigation of the hypothesized convergence. ERK2 activation was measured in four states of activation, within 10 min of application: control slices, 10 µM NMDA, 10 µM dopamine, and NMDA+dopamine, both at 10 µM. The results suggest a significant and clear convergence of the effects of fast and slow neurotransmission upon ERK2 activation (Fig. 4).

Various MAPKs share similar upstream signaling pathways, especially ACs. In order to explore whether this readout for convergence is unique to ERK1/2 or is shared with other MAPKs, we further analyzed possible convergent activation of p38 and JNK. Both p38 and JNK exhibited similar significant activations following application of either NMDA or dopamine separately. However, the impressive converging effect that was found with ERK2, was not detected with the other MAPK's. Thus, the converging signaling pathways are unique to each pattern of activation and are not general. A similar pattern of differential activation of the various MAPK cascades in response to other types of stimulations (Gi- and Gq-coupled receptors) has been detected in neuronal cell culture [42].

As consequence to these findings, the question raised is where, or on which molecule/s, the convergence is taking place. Previous research has shown that there are known interactions between NMDA and dopamine receptors [58], [59], which makes such a convergence at the receptor level feasible.

Hence, we conducted the dopamine and NMDA applications (each agent alone) with antagonists, while using the ERK2 activation as metabotropic readout. Surprisingly, we found that dopamine induced ERK2 activation is dependent upon the NMDA receptor but not vice versa (Fig. 4A, B). In other words, dopamine activates ERK2 through the NMDA receptor.

This finding coincides with other publications [28], [29], [30], that suggest that the actual converging site is the NMDA receptor, and among the various MAPK cascades, ERK2 is the molecule that expresses this convergence, though other signaling cascades can not be excluded. Another supporting evidence for such converging interaction upon the NMDA receptor can be drawn from the resemblance of the two ERK2 activation kinetics patterns: the low doses co-application of NMDA and dopamine and that of the NMDA high dose application. Both applications resulted in a similar ERK2 activation as previously reported. The actual mechanism of that converging interaction needs further investigation, and might be mediated directly at the level of the receptors.

Due to our intent to explore the question of signals convergence as close as possible to the physiological state, we did not differentiate in our current study between the D1 and the D2 dopamine receptors, and such differentiation should be further investigated.

In order to further explore the resemblance between strong NMDA and co-application of weak NMDA and dopamine inputs on cellular measurements, we have analyzed electrophysiologically the fEPSP following these chemical stimulations. In accordance with the biochemical findings, similar results were shown by the electrophysiological essay. The effect of low dose dopamine application resulting with a weak and sustained LTP is totally inhibited by employing APV concurrently (Fig. 5C). Hence, suggesting that the dopaminergic effect upon synaptic transmission is conveyed through the NMDA receptor. Indeed, strong and transient depression was observed following application of high dose of NMDA (Fig. 5A) and co-application of low doses of NMDA and dopamine (Fig. 5D) but not with low doses of either NMDA (Fig. 5B) or dopamine (Fig. 5C) alone. This strong short-term depression is totally NMDA dependent, as APV application inhibits any change in synaptic response. Furthermore, not only the dopamine “enhancing” effect of the depression is abolished by APV, but neither low dose NMDA nor low dose dopamine effects are present. Once again, the erase of even the potentiative dopamine alone effect, strongly support the notion that it is mediated via the NMDA-R.

These results, of a fast short term depression of the fEPSP with a gradual return to baseline, coincide with former findings reported with NMDA application on hippocampal cell culture [60] [61]. These protocols induced a rapid and transient internalization of α-amino-5-hydroxy-3-methyl-4-isoxazole propionic acid (AMPA) receptors, which is NMDA dependent, quite similar to the electrophysiological kinetics received in our experiments (Fig. 5A). Our findings correspond with these results, also in the proposed molecular mechanism for the short-term depression, considering that the major component of the fEPSP is contributed by the AMPA receptors. As shown in our results, both high dose of NMDA and co-application of low doses of NMDA and dopamine induced GluR1 withdrawal from the synaptosomal fraction (Fig. 7), suggesting for a resemblance also in the molecular mechanisms involved for this synaptic depression. The AMPA-R internalization, observed in hippocampal cell culture is calcineurin dependent [61]. Interestingly, it was recently shown that AC (not AC1 or 8) is activated by the same calcineurin [31]. Furthermore, NMDA induces this particular AC activation via calcineurin, and APV inhibits its induction. It is not clear currently, what are the relationships between these two NMDA and calcineurin dependent processes?

**Figure 8:**
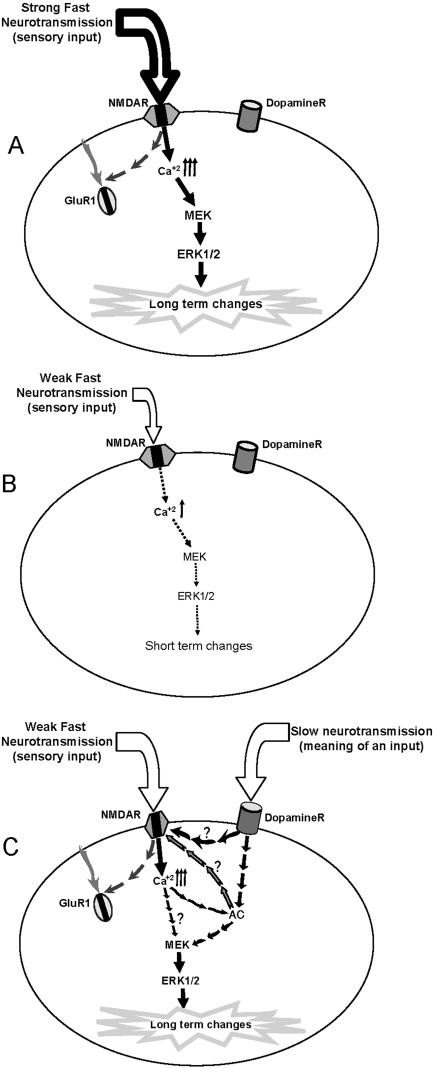
An illustration of possible fast and slow neurotransmission convergence. A. shows that a strong input of fast neurotransmission (which means either large magnitude, long duration, or optimal spacing) causes GluR1 insertion and strong activation of ERK1/2, which may lead to increased protein synthesis and long-term changes (e.g., Late-LTP or Long Term Memory). B. shows that a weak input of fast neurotransmission (weak means small magnitude, too short duration, or non-optimal spacing) would lead to weak ERK1/2 activation, and would result in only short-term changes if any. C. shows that though the fast neurotransmission input is weak (as most ordinary daily physiological input is), the convergence of a slow neurotransmission (e.g., dopamine) upon the fast neurotransmission receptor induces GluR1 insertion and strong and fast ERK2 activation, thus gives it another meaning, and consequently causing long-term changes. The process is NMDA-R dependent and can be mediated via the activation of different types of AC's.

In the present study we demonstrated that among MAPKs, ERK2 serves as coincidence biochemical readout of fast and slow neurotransmission of information in the mature brain. Thus ERK2 has the potential to compute information from fast and slow neurotransmissions. Nevertheless, other molecules might be good candidates for conveying similar coincident detection readout. Moreover, this metabotropic effect of both NMDA and dopamine are dependent on the NMDA receptor itself, and the receptor itself serves as the point for convergence and integration of the different stimuli. In a similar manner to the biochemical results, we observed parallel synaptic modifications following strong NMDA application and co-application of dopamine and NMDA. We proposed that long term neuronal modifications, manifested by ERK activation, GluR1 internalization or synaptic depression, can be achieved via two different inputs. The first one is a prolonged and strong NMDA activation and the second one is prolonged and weak co-activation of NMDA and dopamine (Fig 8). These neuronal mechanisms may underlay heterosynaptic mechanisms subserving learning process (Fig. 8). Moreover, they give new molecular framework to the proposed malfunction in dopaminergic and glutamatergic neurotransmissions and the interactions between the two, underlying schizophrenia, especially the negative symptoms and the cognitive deficits involved. It is conceptualized that the negative symptoms and cognitive deficits in schizophrenia are related to either hypodopaminergic transmission in the frontal cortex [62], or to a decreased NMDA receptor functioning [63], and treatments tailored in that direction are becoming favorable [64]. The molecular findings discussed here point to the converging interaction between the two pathways, and might play a role in the understanding of the schizophrenic pathophysiology and treatment.

Further research is needed to identify the subtypes of dopamine receptors involved in the process, the inter-receptorial mechanism of such convergence, the spatial distribution within individual neurons, and the neuronal events determined by such convergence.

## Materials and Methods

### Subjects

Adult male Sprague-Dawley rats, weighing 300–350 g (Harlan, Jerusalem, Israel), were housed individually and maintained on a 12/12-h light/dark cycle. The experiments were approved by the Institutional Animal Care and Use Committee (Haifa University and University of Wisconsin), and adequate measures were taken to minimize pain or discomfort, in accordance with the guidelines laid down by the NIH, regarding the care and the use of animals for experimental procedures.

### Hippocampal slice preparation

After decapitation the brain was immediately immersed in cold (4°C) carboxygenated (95% O_2_, 5% CO_2_) Artificial Cerebro Spinal Fluid (ACSF [in mM]: 124 NaCl, 5 KCl, 1.2 MgSO4, 1.2 NaH_2_PO_4_, 26 NaHCO_3_, 10 D-glucose, 2.4 CaCl), and after about 120 s both hippocampi were dissected out in a plate filled with cold (4°C) ACSF on ice. The hippocampi were put on a cooled stand of a McIlwain tissue chopper TC752 (Campden Instruments Ltd, UK), cut into 400-µm slices, and then put back into a chamber filled with carboxygenated cold (4°C) ACSF.

The slices were transferred to a holding chamber for about 20–30 min, to reach room temperature, and were then transferred to a six-chamber pharmacological instrument, designed to our specifications by Scientific Systems Design Company (Ontario, Canada). All of the slices tested in any one experiment (i.e., in all six chambers) were produced by the same procedure from the same rat.

### Reagents

N-methyl-D-Aspartate (NMDA, 10 µM and 100 µM) was purchased from Tocris (Ellisville, Mo, USA). Dopamine (10 µM and 100 µM), ascorbic acid (free acid) (1 mM), glycine (10 µM), TTX (1 µM), NMDA antagonist - 2-amino-5-phosphonovalerate (APV, 50 µM), MEK inhibitor U0126 (30 µM) were all from Sigma (Steinheim, Germany).

NMDA (10 µM and 100 µM) was always co-applied with 10 µM glycine, and dopamine (10 µM and 100 µM) was always co-applied with 1 mM ascorbic acid. This was done to ensure the conservation of dopamine activity in the course of the experiment, and also to protect the hippocampal slices against the possible cytotoxic effect of dopamine. Ascorbic acid at 1 mM has been shown not to affect MAPK activity [19], [65].

p44/42 MAP kinase antibody (1∶1000), rabbit polyclonal; phospho-p44/42 MAP kinase (Thr202/Tyr204) antibody (1∶500), rabbit polyclonal; p38 antibody (1∶500), rabbit polyclonal; phopho-p38 (Thr180/Tyr182) antibody (1∶500), rabbit polyclonal; Jnk antibody (1∶500), rabbit polyclonal; phospho-Jnk (Thr183/Tyr185) antibody (1∶500), rabbit polyclonal were purchased from Cell Signaling (Beverly, MA, USA). β-actin antibody (1∶3000), goat polyclonal; PSD95 antibody (1∶500), mouse monoclonal were purchased from Santa-Cruz Biotechnology (Santa Cruz, CA, USA).

Goat anti-mouse (IgG) horseradish peroxidase (HRP) conjugated was from Jackson Immunoresearch (West Grove, PA, USA). Goat anti-rabbit, (IgG) HRP conjugated and the Enhanced Chemiluminescence (ECL+) Kit were from Amersham (Piscataway, NJ, USA).

### Pharmacological chamber handling and manipulation

The hippocampal slices were heated to 32°C and were kept in the chamber for 5 h before any pharmacological intervention. Each chamber contained four slices. The slices were perfused with heated and carboxygenated ACSF via a Model MP3 peristaltic pump (Gilson, France), at a rate of ∼2 mL min^−1^. The chamber space was carboxygenated and humidified. The chamber was an interface type, and the slices were placed upon a lens paper. Within each experiment, one chamber was used as a “positive control” for the quality of the slices by infusing the chamber with NMDA 100 µM together with glycine 10 µM, which is known to induce a strong ERKII activation. Only experiments that showed an arbitrarily chosen ERKII activation level of at least 25% were considered. Moreover, viability of the slices after this prolonged incubation period was occasionally tested by electrophysiological means: slices were transferred to an electrophysiological chamber, and the response from the stratum-radiatum in the CA1 region was recorded, while the Schaffer collateral pathway was stimulated. Viability was verified by the presence of a fEPSP, and facilitation of fEPSP.

There was no pooling of results from different comparing protocols experiments which shared a part of the same stimulation protocol. Each of the comparative experiments and analysis used a new batch of slices that came from the same rat via the same preparation process.

### Preparation of samples

At several time points following the pharmacological manipulation, hippocampal slices were removed from the pharmacological chamber and snap frozen on dry ice. After freezing, slices were homogenized in SDS sample buffer as previously described [66]. Four slices from each chamber were combined as two pairs and the two slices of each pair were homogenized as a single sample, so that each chamber yielded two samples (n = 2).

### Preparation of Synaptoneurosomal fraction

The protocol was adopted from Quinlan [67] and is similar to the protocol used in [68]. In brief: four hippocampal slices (from each chamber) were snap-frozen on dry ice, homogenized in a teflon/glass 2.5 ml tissue grinder, with 1.5 ml of homogenization buffer [10 mM HEPES, 2 mM EDTA, 2 mM EGTA, 0.5 mM DTT, 1% phosphatase inhibitor cocktail (Sigma) and 1% protease inhibitor cocktail (Sigma)]. An aliquot of the homogenized tissue (50 µl) was retained and mixed with 50 µl SDS sample buffer ×2 (Total fraction). The remaining material was passed once through a 100 µm filter and once through a 5 µm filter (Millipore), attached to a 2 ml syringe. The homogenized tissue was centrifuged at 3200 rpm for 10 min at 4°C. The pellet contained the synaptoneurosome fraction, while non-specific material remained in supernatant. SDS sample buffer ×2 was added to both the pellet and supernatant fractions, the samples were boiled for 5 min, and stored at −20°C.

### Quantification

Quantification of immunoblots was performed with a CCD camera, Model XRS (Biorad, Italy). Each sample was measured relative to the background. Phosphorylation levels were calculated as the ratio between the readings from the antibody directed against the phospho-proteins and those from the antibody directed against the phosphorylation-state-independent form of the proteins. Phosphorylation of a protein (phospho-protein/protein) is expressed as the ratio between the readings from the pharmacologically manipulated slices and those from the control slices within the same batch of slices in the same experiment. A value of 1 indicates no difference in protein phosphorylation, whereas values different from 1 indicate an increase or decrease in phosphorylation. The value multiplied by 100 represents the percentage change in protein phosphorylation.

### Electrophysiology

Transverse hippocampal slices (400 µm) were isolated with a Vibratome (Vibratome, St. Louis, MO) in ice cold cutting saline (CS [in mM]: 110 Sucrose, 60 NaCl, 3 KCl, 1.25 NaH_2_PO_4_, 28 NaHCO_3_, 0.5 CaCl_2_, 7 MgCl_2_, 5 Glucose, 0.6 Ascorbate). Slices were allowed to recover for 45 min at room temperature in 50∶50 CS∶ACSF. After initial recovery, slices were placed in an interface chamber (Fine Science Tools, Foster City, CA) and maintained at 30°C in ACSF (1 mL/min). Slices were allowed to recover for an additional 60 min on the electrophysiology rig prior to experimentation. Bipolar stimulating electrodes (92:8 Pt:Y) were placed at the border of Area CA3 and Area CA1 along the Schaffer-Collateral pathway. ACSF-filled glass recording electrodes (1–3 MΩ) were placed in stratum radiatum of Area CA1. Basal synaptic transmission was assessed for each slice by applying gradually increasing stimuli (0.05–1.5 µA, 0.5–15 V, A-M Systems model 2200 stimulus isolator, Carlsborg, WA) and determining the input:output relationship. All subsequent stimuli applied to slices was equivalent to the level necessary to evoke a fEPSP that had a left slope which was 50% of the maximal left slope that could be evoked. Synaptic efficacy in response to treatment of slices with various neurotransmitters and drugs was continuously monitored (0.05 Hz). Sweeps were averaged together every 2 min. In experiments where paired-pulse facilitation was measured, two stimuli administered 50 msec apart were applied every 10 min while monitoring synaptic efficacy. fEPSPs were amplified (A-M Systems Model 1800) and digitized (Digidata 1322B, Molecular Devices, Sunnyvale, CA) prior to analysis (pClamp, Molecular Devices).

### Statistical Analysis

The results are expressed as means±SEM, and the number of samples. For statistical analysis one-way ANOVA was applied. For post-hoc comparisons, Fisher's LSD test was used. Significance for all tests was set at an α level of 0.05. Analysis of electrophysiology was performed using either a 1-way (Fig. 5 A,B,D,E) or 2-way ANOVA (between: treatment, within: time; Fig.5C,F). Post-hoc analysis was performed using the method of Bonferroni. Paired-pulse facilitation (Supp Fig. 1) was analyzed using a 1-way ANOVA. Significance for all tests was set at p≤0.05.
